# Monitoring of Stimulus Evoked Murine Somatosensory Cortex Hemodynamic Activity With Volumetric Multi-Spectral Optoacoustic Tomography

**DOI:** 10.3389/fnins.2020.00536

**Published:** 2020-06-03

**Authors:** Benedict Mc Larney, Magdalena Anastasia Hutter, Oleksiy Degtyaruk, Xosé Luís Deán-Ben, Daniel Razansky

**Affiliations:** ^1^Faculty of Medicine, Technical University of Munich, Munich, Germany; ^2^Institute for Biological and Medical Imaging, Helmholtz Center Munich, Munich, Germany; ^3^Faculty of Medicine and Institute of Pharmacology and Toxicology, University of Zurich, Zurich, Switzerland; ^4^Institute for Biomedical Engineering and Department of Information Technology and Electrical Engineering, Eidgenössische Technische Hochschule (ETH) Zürich, Zurich, Switzerland

**Keywords:** optoacoustics, hemodynamics, somatosensory, cortex, initial-dip

## Abstract

Sensory stimulation is an attractive paradigm for studying brain activity using various optical-, ultrasound- and MRI-based functional neuroimaging methods. Optoacoustics has been recently suggested as a powerful new tool for scalable mapping of multiple hemodynamic parameters with rich contrast and previously unachievable spatio-temporal resolution. Yet, its utility for studying the processing of peripheral inputs at the whole brain level has so far not been quantified. We employed volumetric multi-spectral optoacoustic tomography (vMSOT) to non-invasively monitor the HbO, HbR, and HbT dynamics across the mouse somatosensory cortex evoked by electrical paw stimuli. We show that elevated contralateral activation is preserved in the HbO map (invisible to MRI) under isoflurane anesthesia. Brain activation is shown to be predominantly confined to the somatosensory cortex, with strongest activation in the hindpaw region of the contralateral sensorimotor cortex. Furthermore, vMSOT detected the presence of an initial dip in the contralateral hindpaw region in the delta HbO channel. Sensorimotor cortical activity was identified over all other regions in HbT and HbO but not in HbR. Pearson’s correlation mapping enabled localizing the response to the sensorimotor cortex further highlighting the ability of vMSOT to bridge over imaging performance deficiencies of other functional neuroimaging modalities.

## Introduction

Imaging the brain with high resolution, both at functional and anatomical levels, and in real time is essential for unraveling the mysteries of how we think and act. In the last few decades, vast efforts have been directed to the development of new approaches for scalable imaging of brain activity (Markram et al., 2011; Insel et al., 2013). The brain is known to consist of billions of cells, predominantly neurons, glia, and endothelial cells, where neuronal communication is achieved through electrical signals transmitted across a hugely dense network. Monitoring these orchestral interactions at a global level in mammalian brains remains a major challenge in neuroscience and current research efforts are transitioning to enable imaging the functioning brain in its entirety as opposed to focusing on single or small numbers of cells (Fornito et al., 2015; Mott et al., 2018). Current limiting factors of this transition are the need for high spatio-temporal resolution across the entire brain, non-invasivity and specificity of the recorded responses (He et al., 2011).

Neuronal activity is highly demanding in terms of energy consumption (Herculano-Houzel, 2011). The brain vasculature provides neurons with a continuous supply of oxygen and nutrients to satisfy the local demand in activated areas (Buxton et al., 1998; Yablonskiy et al., 2013) resulting in rapid hemodynamic changes that are known to be linked to neuronal activation via neurovascular coupling (Filosa and Blanco, 2007). Functional magnetic resonance imaging (fMRI) is the predominant method used to image these changes (Bosshard et al., 2010; Yablonskiy et al., 2013). However, the method’s spatial and temporal resolution is limited while quantification of hemodynamic responses further relies upon theoretical models to extract reduced hemoglobin (HbR) levels from the measured blood oxygenated level dependent (BOLD) signals, the relationship of which to neuronal activity is still not fully understood (Arthurs and Boniface, 2002; Iannetti and Wise, 2007; Logothetis, 2008).

Recently, alternative functional brain imaging approaches based on optical and ultrasound methods have shown great promise. In particular, optical methods capable of imaging hemodynamic responses include optical intrinsic signal imaging (OISI), laser speckle imaging (LSI) and diffusion optical tomography (DOT) (Culver et al., 2004; Kazmi et al., 2015; Reisman et al., 2017). These methods enable enhanced contrast, increased speed and significantly reduced costs with respect to fMRI. However, they are strongly affected by the scattering of light within biological tissues, which limits their applicability to superficial layers of the cortex (Hielscher, 2005), even for state of the art approaches that do not require removal of the scalp or skull (Reisman et al., 2017). Due to weak scattering of ultrasound waves in soft tissues, functional ultrasound (fUS) is able to achieve imaging of deep brain areas with diffraction-limited ultrasonic resolution but skull removal is often required for high resolution studies. Furthermore, its contrast primarily relies on cerebral blood flow (Macé et al., 2011; Deffieux et al., 2018), making it insensitive to changes in other relevant hemodynamic parameters occurring during brain activation, such as oxygenation changes.

Multi-spectral optoacoustic tomography (MSOT) has recently emerged as a powerful approach for studying brain activity in rodents (Ovsepian et al., 2019b). The endogenous contrast provided by the optical absorption of blood enables mapping multiple hemodynamic parameters (Hu and Wang, 2010; Li et al., 2010; Gottschalk et al., 2015, 2017; Yao et al., 2015), while the penetration depth and resolution are comparable to those achieved with fUS. MSOT systems facilitate real-time volumetric (three-dimensional) imaging with scalable spatial resolution and field of view (FOV) not easily achievable with other optical modalities such as OISI, DOT or LSI. Therefore, MSOT effectively bridges the gap between cellular resolution microscopy on a sub-millimeter scale and macroscopic neuroimaging observations with poor spatial resolution (Dean-Ben et al., 2016, Deán-Ben et al., 2017; Ovsepian et al., 2019a). To date, various stimulation paradigms, including optogenetic, paw and whisker, have been imaged with MSOT. These enabled readily detecting functional activation via hemodynamics with anatomic landmarks (Hu and Wang, 2010; Olefir et al., 2019; Ovsepian et al., 2019a, b). Previous studies have shown capabilities of non-invasive imaging through the skull and scalp linking epileptic seizure activity to electrophysiological activity (Gottschalk et al., 2017). The fast imaging speed and spectroscopic differentiation capacity have further enabled imaging of calcium dynamics *in vivo* (Dean-Ben et al., 2016; Gottschalk et al., 2019).

Despite its promising imaging performance showcased in initial studies, the utility of MSOT for studying the processing of peripheral inputs has only been achieved at small cerebral FOVs and its application to the whole brain has so far not been quantified (Olefir et al., 2019; Ovsepian et al., 2019a). In this work, we employ a state-of-the-art volumetric MSOT (vMSOT) system providing five dimensional (5D, three-dimensional, real-time and multi-spectral) imaging capabilities in order to map cerebral hemodynamic changes in mice in response to a standard electrical paw stimulation paradigm commonly used in fMRI (Schroeter et al., 2014). Electrical paw stimulation has the added advantage of being applicable to a wide variety of model organisms, providing a robust and replicable method of investigating brain responses to peripheral stimuli. As opposed to fMRI and other conventional neuroimaging methods, high-resolution imaging was achieved over the entire isocortex. This enabled mapping of real time simultaneous responses of all hemodynamic components (oxy- and deoxy-hemoglobin, blood volume) to peripheral stimuli across multiple brain regions. The high contrast of the vMSOT images corresponding to visible optical wavelengths facilitates detecting signals from small cortical arterioles and venules, which are known to undergo greater changes than larger vessels during the hemodynamic response (Ma et al., 2016a; He et al., 2018).

## Materials and Methods

### vMSOT Imaging

**FIGURE 1:**
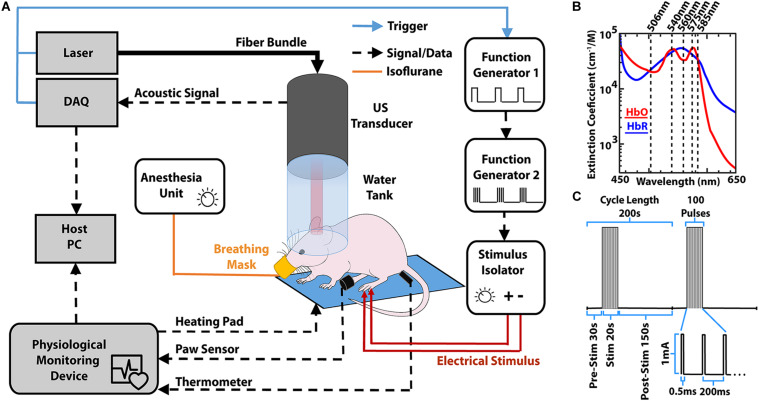
**(A)** Lay-out of the experimental set-up for inducing and monitoring hemodynamic responses in mice using volumetric multi-spectral optoacoustic tomography (vMSOT). Both the image acquisition and paw stimulation are synchronized via the laser trigger. The physiological monitoring device allows consistent monitoring of the mouse to ensure anesthesia is maintained at 1–1.5% isoflurane levels. DAQ, Data Acquisition Unit. **(B)** Molar extinction coefficient for HbO and HbR in the visible spectrum of light (Jacques, 2013a). Superimposed are the wavelengths that were used for imaging. **(C)** Paw stimulation paradigm similar to that used in fMRI to elicit the hemodynamic response (Schroeter et al., 2014).

The imaging set-up used in the experiments has been previously described in detail (Gottschalk et al., 2019). In summary, the 512-element hemispherical transducer array (Imasonic SaS, Voray, France) has a central aperture through which a fiber optic bundle is placed (CeramOptec GmbH, Bonn, Germany). The bundle is coupled to a short-pulsed (<10 ns) optical parametric oscillator laser capable of sweeping wavelengths from 420 to 680 nm (Innolas Laser GmbH, Krailing, Germany) on a per-pulse basis. Visible wavelengths of light were chosen for an optimal signal-to-noise ratio (SNR) of the cortical vasculature. The pulse repetition frequency (PRF) of the laser was set to 20 Hz, with five wavelengths (506, 540, 560, 575, and 585 nm, see Figure 1B) being repeated in succession to efficiently cover the relatively slow hemodynamic changes for a trial length of 200 s with multiple repeats (see section “Stimulation Paradigm”). The vMSOT images featuring endogenous blood contrast were processed via linear spectral unmixing to isolate the oxygenated hemoglobin (HbO), reduced hemoglobin (HbR) and total hemoglobin (HbT) components using the spectral absorption profile of hemoglobin (Jacques, 2013a). Note that the linear unmixing approach is prone to spectral coloring artifacts when rendering concentrations of exogenous agents and blood oxygenation values in deep tissues (Cox et al., 2012; Tzoumas et al., 2016; Tzoumas and Ntziachristos, 2017). In our case, the light fluence distribution at the mouse cortex, where hemodynamic changes were mapped, was assumed to be approximately the same for all wavelengths due to the relatively superficial imaging depth, in a way that linear unmixing provides sufficiently accurate readings. In addition, no quantitative sO_2_ values were extracted in our study, which solely relied on relative readings of the individual HbO and HbR components (Laufer et al., 2005; Li et al., 2008; Cox et al., 2012).

### Animal Preparation and Paw Stimulation

Experiments were performed in 4–6-week-old female athymic nude *FoxN1*^*nu*^ mice (*n* = 4, Envigo, New Jersey, United States, *Foxn1*^*nu*^ 069, weight range 16–20 g). Mice were housed in transparent cages (GM500 IVC Green Line, Tecniplast Deutschland GmbH, Hohenpeissenberg, Germany) which in line with institutional guidelines could house up to 5 mice per cage. The housing ensured a 12 h day and night system with lights turning on at 8 am. Temperature was controlled at 24 ± 1°C with a relative humidity range between 40 and 60%. Animals had access to food and water *ad libitum*. This study was carried out in full compliance with the institutional guidelines of the Institute for Biological and Medical Imaging and with approval from the Government District of Upper Bavaria. The mice were anesthetized by placing them in a chamber containing isoflurane at a concentration of 3% v/v isoflurane in 100% O_2_. Subsequently, they were transferred to a stereotaxic head holding frame (Narishige International Limited, London, United Kingdom) and ultrasound gel (Aquasonic Clear Ultrasound Gel, Parker Laboratories Inc., NJ, United States) was applied on top of the scalp of the animals after being cleaned of any dirt.

The imaging equipment comprising of a water tank and a hemispherical transducer array was then placed above the head of the mice as schematically depicted in Figure 1A. Physiological conditions [heart and breathing rates, temperature and peripheral capillary oxygen saturation (SpO_2_)] were monitored and maintained using an *ad hoc* apparatus (PhysioSuite^®^, Kent Scientific, Torrington, CT, United States). During all experiments the heart rate of the mice was maintained at ∼400 beats per minute (bpm). The PhysioSuite apparatus provided real time information of the heart bpm and this was continually monitored during data acquisition. The anesthesia level was reduced to between 1 and 1.5% v/v isoflurane in 100% O_2_ during the measurements, whilst monitoring heart pace and altering anesthesia depth to maintain a heart rate of ∼400 bpm. Sufficient anesthesia depth was ensured by testing the toe pinch reflex of the non-stimulated paw (both before and after trials). Following data acquisition, all mice were removed from the head holder and allowed to recover from anesthesia. No adverse effects such of the experiment were noted in any of the mice.

### Stimulation Paradigm

Electrical stimulation was performed by placing two stainless steel electrodes beneath the skin of one of the hind paws of the mouse. The electrodes were connected to a stimulus isolator (Model A365R, World Precision Instruments, Sarasota, FL, United States) that delivered a train of 100 pulses (0.5 ms, 1 mA) at 5 Hz for 20 s (Schroeter et al., 2014). The total trial time was 200 s, with a 30 s baseline (pre-stimulation), 20 s of stimulation and 150 s of recovery (post-stimulation, see Figure 1C). For each measurement, the trial was repeated consecutively two times. Measurements were also carried out in the same fashion but without applying any electrical stimulation for a single trial prior to the stimulation experiment (negative control) where no activation was detected, as shown in Supplementary Figure S3.

### Data Analysis

**FIGURE 2:**
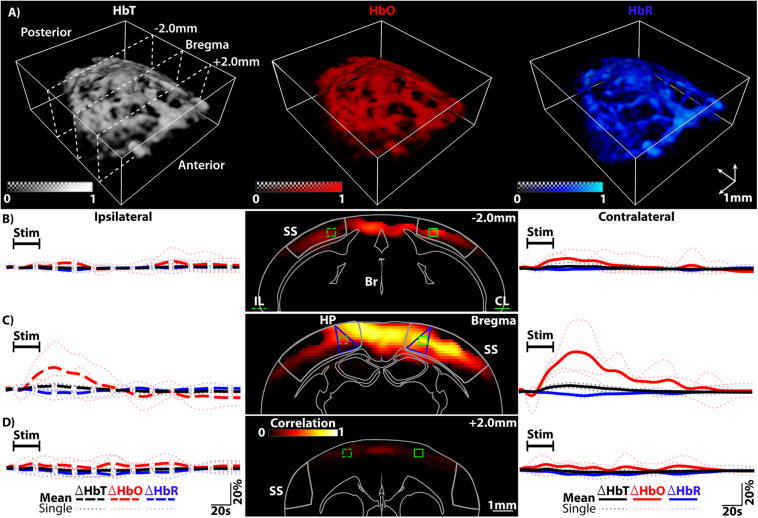
**(A)** Spectrally unmixed distributions of HbT, HbO, and HbR estimated with vMSOT including slice locations. **(B)** Left, the ipsilateral (IL) traces (large dotted lines) for HbT, HbO, and HbR from the IL ROI highlighted in dotted green in the middle image. Middle, Pearson’s correlation mapping of the activity [-2.0 mm from Bregma – primarily visual cortex (VC) regions]. Right, the contralateral (CL) traces from the same area (solid green ROI). The SS area is outlined. **(C)** Left, IL traces for HbT, HbO, and HbR from the IL ROI highlighted in dotted green in the middle image [at Bregma – primarily hind paw (HP) somatosensory regions]. Middle, Pearsons correlation mapping highlighting the extent of activity. Right, the CL traces from the same area (solid green ROI). Seed voxels located within the CL-HP ROI were used for correlation mapping. HP regions of the SS cortex are highlighted (blue lines) as well as the SS cortex (gray outline). **(D)** Left, traces for HbT, HbO and HbR from the IL ROI highlighted in dotted green in the middle image (+2.0 mm from Bregma – primarily somatomotor cortex regions). Middle, Pearsons correlation mapping at this location. Right, CL traces from the same area (solid green ROI). Legend – IL, Ipsilateral; CL, Contralateral; HP, Hindpaw region of somatosensory cortex; SS, Somatosensory cortex; Br, Brain Outline. The average response from all trials from each mouse is plotted as a lightly dotted line in all cases. The bold line represents the averaged response from all mice.

All data was reconstructed offline using a graphics processing unit (GPU) implementation of the back-projection algorithm, as previously described (Ozbek et al., 2013). In brief, the signals were band-pass filtered between 100 kHz and 6 MHz to remove both low frequency bias and high frequency noise components. The reconstructed images were normalized on a frame by frame basis to compensate for laser fluctuations as well as for the wavelength-dependent energy of the pulse. This was achieved by constantly measuring the optoacoustic signal from of a ∼150 μm sphere (Cospheric LLC, Santa Barbara, CA, United States) located on the foil separating the water tank from the head of the mouse. Following unmixing, the relative changes of each component (%Δ) were calculated. This was done by subtracting the baseline image, estimated as the average of the first 30 s of the trial, from the image at each time point and subsequently normalizing by such baseline. Stimulation trials were recorded consecutively from each mouse with responses being detected in all cases. Coronal slices were chosen within the approximate locations of visual (VC), somatosensory (SS) and somatomotor (SM) cortical regions in relation to the anatomical landmark bregma and within the isocortex, for each mouse (−2.0 mm from bregma – VC, bregma – SS and + 2.0 mm – SM, respectively, see Figure 2A) and Supplementary Figure S2A) (Takamasa et al., 2001; Oh et al., 2014; Paxinos and Franklin, 2019). Using available atlases, the coronal slices were compared to known regions and ROIs (5 × 5 × 3 voxels) were chosen for analysis, including the primary SS region corresponding to lower limbs (S1HL or SSp-II) (Oh et al., 2014; Paxinos and Franklin, 2019). The mean value from all voxels within the ROI was used to compute traces. Single trials were analyzed and the response from each mouse for HbO, HbR, and HbT was averaged into a single representative trace for each mouse. Finally, the averaged responses from all mice for each component and respective region were averaged to determine the population response from the stimulation and non-stimulation trials.

Statistical analysis was carried out via custom code in Matlab (2018b, Mathworks, MA, United States) on the trace data from both stimulation trials from all mice (8 trials in total). The code automatically sorted through each trace to find its peak activation percentage (P%), peak dip percentage (Dip%) and the times taken to reach peak activation (TTP), the dip (DTTP) and decay time (DeT). All times were calculated on the 10th and 90th percentile change. Statistical significance was determined via a Wilcoxon signed-rank test where *p* < 0.05 was determined as statistically significant (Liao et al., 2012). Note that for HbO and HbT a dip would constitute a downward trend before reaching peak activation and for HbR a dip would constitute an upward trend before reaching peak activation. Each contralateral (CL) region was compared to its ipsilateral (IL) counterpart and finally the region of the SS known to correspond to contralateral hindpaw (CL-HP) stimulation region was tested for significance over all other regions (Takamasa et al., 2001; Oh et al., 2014; Paxinos and Franklin, 2019).

### Functional Cortical Mapping

Pearson’s correlation coefficient mapping was carried out on the HbO data (any data likely to include the scalp and skull was removed) to determine the extent of activation (Huppert et al., 2006; Cui et al., 2017). The correlation map of the brain was generated by using the time trace of the CL-HP SS ROI within the activated region and then calculating the time correlation coefficient of this trace to all other voxels in the brain. For correlation mapping, a ROI of 1 × 3 voxels was selected from the previous ROI used to determine the activity traces. Activation data from both trials of each mouse were averaged into a single representative trial. Smoothening was applied to the entire imaged FOV prior to calculating the correlation map using a 3 × 3 × 3 kernel of ones and any data outside the brain was removed via thresholding. To allow for a slight time shift but still correlated signals a permissible lag of ±2 s was included. The maximum value of each correlation coefficient was then chosen to compose a three-dimensional map of correlation values. To visualize the data more clearly the map was thresholded to only include positive correlation values where 0 corresponds to no correlation and 1 corresponds to maximum correlation.

### Hemodynamic Imaging

The set-up to both evoke and comprehensively capture numerous components of the hemodynamic response is outlined in Figures 1A–C. The current set-up allows for complete movement of the mouse head (all axes and rotation) to ensure optimal placement. The ability to monitor the heart rate and other physiological states ensured that the heart rate was maintained at ∼400 bpm throughout the experiments whilst also keeping the mouse anesthetized. To ensure synchronization of both the imaging and stimulus paradigm the laser trigger was used to control the entire experiment.

## Results

### Preserved Contralateral Activity

For all experiments the paw stimulation paradigm was carried out as outlined in the methods section. As shown in Figure 2 (A – representative of *n* = 4 mice), vMSOT is readily capable of rendering the HbO, HbR, and HbT components after unmixing the multi-spectral data. The field of view (FOV) covers the cortical regions. For ease of visualization all IL traces are bold dotted lines on the left hand side of Figures 2B–D, left panels and all CL are solid lines on the right (Figures 2B–D, right panels), irrespective of their original orientation. In all cases ROIs were chosen within the VC, SS, or SM regions of the isocortex [Figures 2B–D, middle panels, dotted (IL) and solid (CL) green lines]. Note that blue segmented regions highlight the HP region of the SS cortex. The corresponding time courses for HbO, HbR, and HbT averaged over both trials from each mouse are shown (lighter colored dotted lines, Figures 2B–D), including the average response from all mice (population response, bold lines Figures 2B–D). The traces have been cropped to show 5 s before stimulation, the stimulation window and then the remaining 150 s recovery period. As can be seen in the traces the hemodynamic changes clearly evince that stimulating the paw results in hemodynamic activity across the entire isocortex, with the CL side of the brain undergoing the strongest reaction, especially the CL-HP region.

**FIGURE 3:**
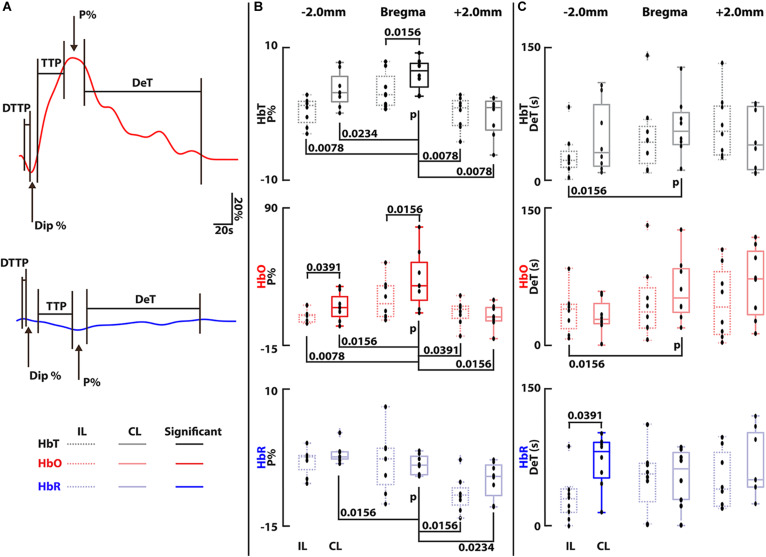
**(A)** Illustration showing how all components were calculated along with a legend for all box plots, these are the same bold traces as shown in Figure 2C, right panel. Components not shown were not significant and are outlined in Supplementary Figure S1. Analysis was carried out on each trace based on the average of the voxels within the ROIs shown in Figure 2 for each mouse (*n* = 4) and each trial (*n* = 2 per mouse, 8 trials in total). **(B)** Peak percentage changes (P%) for all components. Bregma (somatosensory) regions were found to be statistically significant from its L counterpart in both HbT and HbO channels and -2.0 mm (visual cortex) was significant in the HbO channel. No statistical significance could be found between the bregma CL and IL region in the HbR channel. The CL-HP region was found to be statistically significant in HbT and HbO over all other regions. **(C)** Decay Time (DeT) for all components. Statistical significance was found in the HbR channel between the IL and CL -2.0 mm region. The CL-HP region was found to be significant over the IL visual region. Times were calculated based on the 10th and 90th percentile signal change. Outliers are marked by dots marked with a cross. Each black dot represents an individual measurement from all trials from all mice (8 dots per box). Statistical significance was determined in all cases via a Wilcoxon signed-rank test comparing ipsilateral to contralateral regions for each component. *P*-values are shown when significant (*p* < 0.05).

Having extracted numerous activation traces from each trial and each mouse, quantitative evaluation of the detected activations in each region and the CL and IL counterparts was carried out. Five components of the traces from all trials and all mice were investigated: the peak percentage change (P%), the time to this peak (TTP), the decay time (DeT), the dip percentage (Dip%) if seen and the dip time to peak (DTTP) – see Figure 3A. P% and DeT were found to be consistently significant and are shown in Figures 3B,C. Data is plotted with IL traces for each region on the left (dotted lines) and CL regions on the right (solid lines). Each CL region was tested for significance over its IL counterpart and the CL-HP region was tested for significance over all regions (CL and IL). The strongest activity is located within the SS CL-HP region of the brain (bregma – HP regions are outlined by the additional blue triangular line in Figure 2C, middle panel.

The magnitude of the measured ΔHbO, ΔHbR, and ΔHbT responses for the CL-HP SS region (Bregma) achieves a median P% change of 31.4, -3.5, and 6.6% with a TTP of 20.5, 22.6, and 21 s HbO, HbR, and HbT, respectively. These values are in line with previous studies (Adamczak et al., 2010; Yao et al., 2013). In comparison, the IL-HP region only reaches P% changes of 17.4, −2.6, and 3.0% with a TTP of 18.6, 19.25, and 14.4 s for the respective components. These responses are in line with previous studies (Masamoto et al., 2006; Chen et al., 2011; Ma et al., 2016b). Of note is the presence of an initial dip in the HbO channel of both the IL-HP and CL-HP SS traces (Figure 2C, left and right panels). However, in this study there was no statistical significance between these dips, and they did not occur in all cases. The Dip% and DTTP for these dips are summarized in Supplementary Figure S1. The median decay times (DeT) for the CL-HP region was longer in all cases in comparison to the IL-HP region (52.1, 61.5, and 50.25 s vs. 36.75, 55.85, and 38.85 s for HbO, HbR, and HbT, respectively) but statistical significance was only found in the HbT component (Figure 3C). It was also found that the P% of the CL-HP region was statistically significant over not only its IL counterpart but all other regions in both HbT and HbO (Figure 3B). This was not the case for HbR, which failed to show significance over its IL counterpart (Figure 3B). In general, the IL hemisphere of the brain in comparison to the CL side undergoes a less localized and more generic activation in terms of temporal dynamics and extent of the activation across the VC, SS and SM areas (see Figure 3C and Supplementary Figure S1). As expected, recordings without paw stimulation resulted in no activation (see Supplementary Figures S3A–C).

### Functional Mapping of Cortical Activity

To further demonstrate the capabilities of vMSOT to map cortical hemodynamic responses, Pearson’s correlation mapping was carried out on the HbO data. Due to the dynamic range of the delta values seen in the HbO data (up to 65% in one case), the presence of the initial dip and the difficulty in determining CL and IL activity in the HbR and HbT channels, it was determined this would give the most reliable and accurate result for the mapping process. Additionally, vMSOT is unique in its ability to map HbO at such a scale and resolution. Following the correlation mapping process outlined in the methods, a resulting correlation matrix representative of all mice for each region is displayed in Figures 2B–D, middle panels. Using the delta map as a guide, highly activated seed voxels located within the CL-HP SS region were chosen for correlation mapping (Oh et al., 2014; Paxinos and Franklin, 2019). Coronal slices are shown overlaid on the outline of a brain (Oh et al., 2014). The activation is strongest within the CL-HP SS area, yet a clear global response remains that ranges across a dominant section of the VC, SS cortex, and small portion of the SM.

## Discussion

Numerous imaging modalities currently exist to monitor functional hemodynamic changes in the brain. These range from high resolution small FOV microscopy-based techniques to lower resolution macroscopic MRI- and US-based techniques. Closing the scaling gap between single cell neuronal imaging and a network level visualization approaches remains a pressing challenge. The results presented in this study corroborate and complement common BOLD studies using a similar stimulation paradigm (Schroeter et al., 2014). This was carried out with the added benefit of being able to detect more comprehensive functional information (namely, HbO, HbR, and HbT). The characteristics of the hemodynamic responses detected with vMSOT (including P%, TTP, DeT, Dip%, and DTTP shown in Figures 2B–D, 3B,C and Supplementary Figure S1) are generally comparable to those measured with BOLD and optical techniques (Schroeter et al., 2014; Ma et al., 2016b), although modality cross-validation under the same anesthesia, mouse line and stimulation paradigm was outside the scope of the current investigation. It should be noted that the dynamic range of the ΔHbR signal is approximately twice that of BOLD (3% vs. 1.5%). This increase in detection is likely due to the fact that while HbR is a valid paramagnetic agent, its physical properties in terms of light absorption capabilities are likely stronger than its paramagnetic potential (Pauling and Coryell, 1936; Jacques, 2013a). These unique features of vMSOT, achieved by capitalizing on the synergistic combination of strong optical contrast and high diffraction-limited ultrasonic resolution, enables a comprehensive representation of brain metabolism and function and is poised to provide new insights on the understanding of brain activity.

The traces seen for HbO, HbR, and HbT further allude to previously observed hemodynamic changes with HbO overshooting and then undershooting to levels below the baseline after recovery. The presence of an initial dip in the HbO component located in both of the SS CL and IL-HP regions with stronger dynamics on the CL side, a contentious issue in fMRI, further highlights the strength of vMSOT for hemodynamic monitoring (Hu and Yacoub, 2012). However, this study did not find any statistical significance between the CL and IL dips for either Dip% or DTTP (Supplementary Figures S1B,C). Our results show little to no increase in the HbR before it decreases whilst the HbT increase and slow return to baseline corresponds to the dilation of vessels attributed to the increase in total CBV, similar to what is achieved with fUS (Culver et al., 2005). The vMSOT system employed in this work additionally provides anatomical information that can be efficiently used for co-registration with established atlases of the mouse brain and hence facilitate localizing the activated areas (Figures 2B–D, middle panels and Supplementary Figure S2). This was utilized to show that the activity is strongest not only within the CL-HP region (as expected, Figure 2C, right panel) but a diffuse global response was seen across the isocortex including the VC, SS, and SM regions (Figures 2B–D and Supplementary Figures S2A,B). By quantitatively analyzing the traces for all components, all trials and across all regions it was shown that the CL side of the brain showed consistently stronger activation (P%, Figure 3B) than the IL side along with longer TTP values and DeT values (Supplementary Figure S1A and Figure 3C). However, statistical significance could only be determined in the HbO and HbT channel for P% (Figure 3B). Of particular importance is the fact that the P% of the CL-HP SS region was statistically significant from all other brain regions for HbT and HbO (Figure 3B). Future experiments could likely take advantage of this fact to aid in mapping and localizing activated areas, along with other stimulation paradigms e.g., whisker and optogenetic stimulation (Olefir et al., 2019; Ovsepian et al., 2019a, b). Future studies may further aim at investigating how the hemodynamic responses outlined here vary across different lines and sexes within the same model organism and in comparison to other model organisms (Constantinides et al., 2011; Li and Zhang, 2018; Ovsepian et al., 2019a). It should also be noted that the experiments were carried out in a completely non-invasive manner with skull and scalp remaining intact and without the need for exogenous contrast agents, thus highlighting vMSOTs applicability in neuroimaging studies and potential implications for the imaging of small animal disease models.

A potential limitation of the study described in this work stems from the use of isoflurane anesthesia, which may hinder the hemodynamic responses due to its vasodilatory effect (Constantinides et al., 2011). Other anesthesia methods, such as ketamine or urethane, may result in better mapping and a reduced global response than that shown here (Figures 2B–D, middle panels; Kober et al., 2005). However, due to the added dynamic range of all hemodynamic components, vMSOT is readily able to overcome these anesthesia artifacts. This is proven by the fact the while correlation mapping showed widespread activation, this activation was strongest within the CL-HP SS region. Deeper mapping of the brain could be carried out by employing near-infrared (650–1350 nm) light illumination. However, the weaker absorption of blood at these wavelengths will likely lead to a lower SNR of vascular structures (Jacques, 2013a). This is especially true in the case of smaller arterioles and venules that undergo greater hemodynamic changes than larger vessels (Ma et al., 2016a; He et al., 2018). Visible light was then chosen as the more suitable imaging paradigm for mapping the activity within the superficial location of the SS cortex. Due to the recent advent of calcium imaging with vMSOT in the mouse brain and the current limited palette of suitable near-infrared probes the ability to distinguish endogenous contrast over exogenous probes in the visible regime of light is of great importance (Gottschalk et al., 2019).

Having replicated similar results to those seen in fMRI with a similar stimulation and mapping method, future work should aim at using the suggested methodology to study neurovascular and neurometabolic coupling mechanisms. This may be of especial importance in the postnatal development of mice and in neurodegenerative models. The method could be utilized for research on neuro-degenerative diseases such as Alzheimer’s, where the non-invasive aspect could be of particular use in longitudinal studies (Camandola and Mattson, 2017; Yin et al., 2017). The suggested vMSOT platform combines the contrast-related strengths of optical imaging with the penetration depths and high resolution of ultrasound. As shown in Figure 1A, brain imaging with vMSOT is achieved with only an ultrasound gel applied to the scalp, which may facilitate experiments in head-fixed awake mice and simultaneous or consecutive stimulation of multiple paws (Morales-Botello et al., 2012; Vanni et al., 2017; Desjardins et al., 2019). vMSOT also has greater sensitivity to hemodynamic activity than fMRI via the increased dynamic range of delta activity and visualization of multiple hemodynamic components. All in all, vMSOT’s ability to combine functional and anatomical imaging at superior spatio-temporal resolution across the whole mouse cortex is expected to be of significant advantage to neuroimaging.

## Data Availability Statement

The datasets generated for this study are available on request to the corresponding author.

## Ethics Statement

This study was carried out in full compliance with the institutional guidelines of the Institute for Biological and Medical Imaging and with approval from the Government District of Upper Bavaria.

## Author Contributions

BM, MH, and DR conceived the study. BM carried out all experiments. BM and MH carried out the analysis of the data. DR and XD-B supervised the study and data analysis. All authors were involved in writing the manuscript and discussed the results.

## Conflict of Interest

The authors declare that the research was conducted in the absence of any commercial or financial relationships that could be construed as a potential conflict of interest.
