# Supplementary material for: Monitoring of Stimulus Evoked Murine Somatosensory Cortex Hemodynamic Activity With Volumetric Multi-Spectral Optoacoustic Tomography
Source: Front Neurosci. 2020 Jun 3;14:536. doi: 10.3389/fnins.2020.00536 (PMC7283916; doi:10.3389/fnins.2020.00536)
Supplement: Supplementary file 1 [file Data_Sheet_1.pdf]

Monitoring of stimulus evoked murine somatosensory cortex hemodynamic activity with volumetric multi-spectral optoacoustic tomography

Benedict Mc Larney[1,2†‡], Magdalena Anastasia Hutter[2,‡], Oleksiy Degtyaruk[2,3], Xosé Luís Dean Ben[3,4], Daniel Razansky[1,2,3,4]*

[1]Faculty of Medicine, Technical University of Munich, Munich, Germany

[2]Insititute of Biological and Medical Imaging, Helmholtz Center Munich, Neuherberg, Germany

[3]Faculty of Medicine and Institute of Pharmacology and Toxicology, University of Zurich, Switzerland

[4]Institute for Biomedical Engineering and Department of Information Technology and Electrical Engineering, ETH Zurich, Switzerland

[‡]These authors have contributed equally to this work

†Present address: Benedict Mc Larney, Molecular Pharmacology Program, Department of Radiology, Memorial Sloan Kettering Cancer Center, New York, NY, United States

*** Correspondence:**
Daniel Razansky
daniel.razansky@uzh.ch

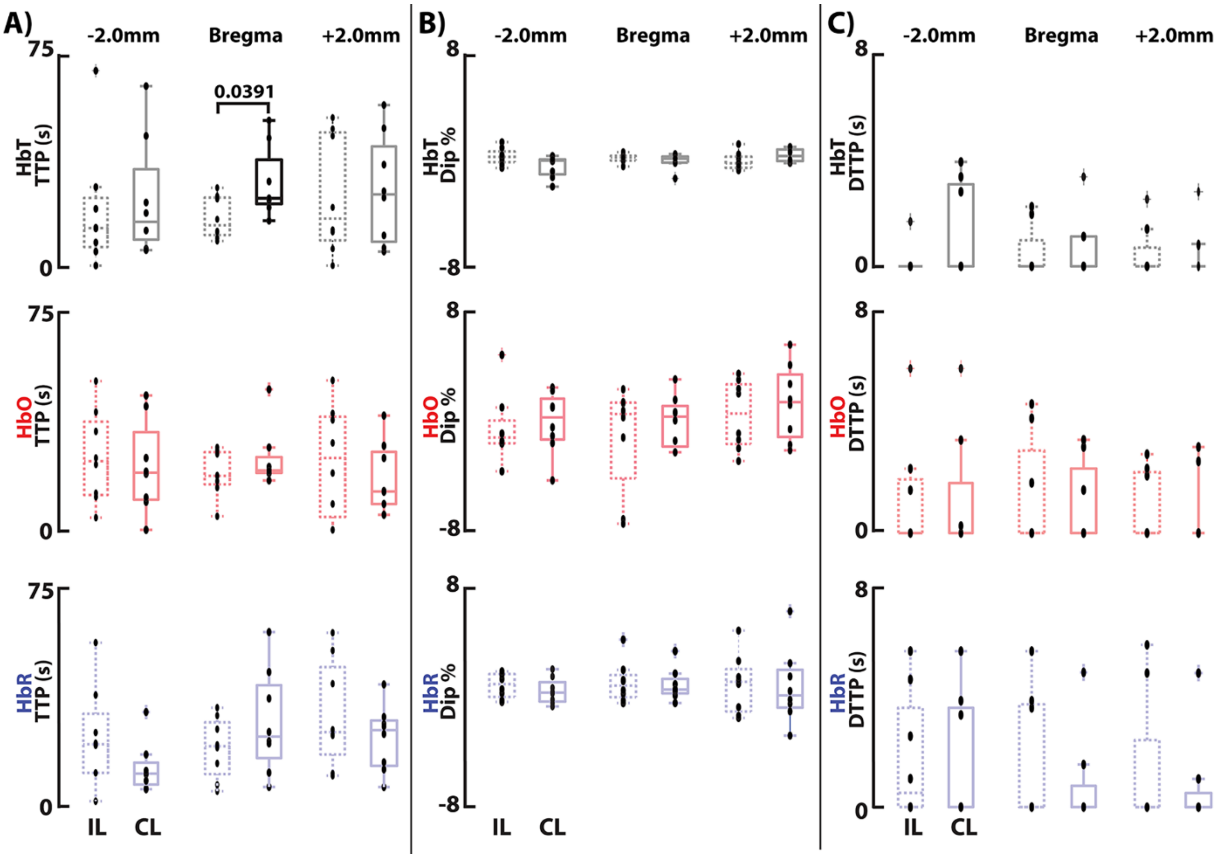

Supplemental Figure 1. A - C) Box plots of all components for each respective region. P values are shown when significant ($p < 0.05$). Analysis was carried out on each trace based on the average of the voxels within the ROIs shown in Figure 2 for each mouse ($n = 4$) and each trial ($n = 2$ per mouse, 8 trials in total). (**A**) Time to peak (TTP) for each component. Statistical significance was only found in the HbT channel between the IL & CL Bregma regions. (**B**) Dip percentage (Dip %) for all regions and components. (**C**) Dip time to peak (DTTP) for each component and region. The median and standard deviation are shown, and whiskers extend to adjacent values. Each black dot represents an individual measurement from all cycles from all mice (8 dots per box). Statistical significance was determined in all cases via a Wilcoxon signed-rank test comparing ipsilateral to contralateral regions for each component.

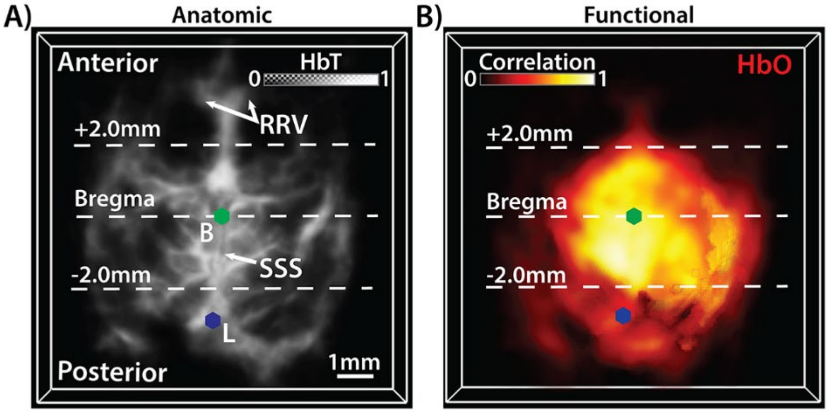

Supplemental Figure 2 A – B) MIPs of 3D Anatomic (HbT) and functional (HbO correlation mapping data). (**A**) Anatomic data (Transverse view, based on vMSOT spectrally unmixed HbT distribution) highlighting the slice locations as shown in Figure 2. Highlighted are anatomical landmarks lambda (L, blue hexagon) and bregma (B, green hexagon). Also highlighted are the superior sagittal sinus (SSS) and rostral rhinal vein (RRV). (**B**) The corresponding MIP of Pearson's correlated HbO data with the anatomical locations identified in A) superimposed. The ability to visualize anatomical landmarks aids in identifying the location of activated areas.

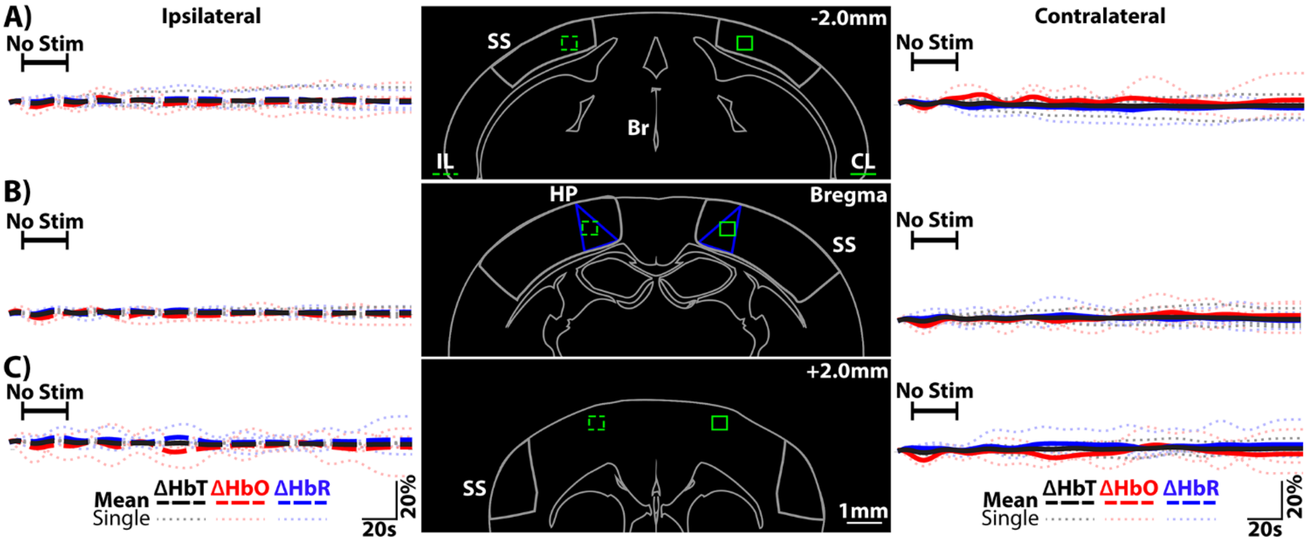

Supplemental Figure 3. A - C) Control data (electrodes inserted but no stimulation applied) for a single cycle from each mouse for HbT, HbO and HbR respectively across all previously analysed brain regions, including anatomical references. These are based on the same mice and ROIs as shown in Figure 2. For ease of comparison, the window in which paw stimulation would be applied is left visible however, no paw stimulation was applied in this case. (**A**) Left, the ipsilateral (IL) traces (large dotted lines) for HbT, HbO and HbR from the IL ROI highlighted in dotted green in the middle image (-2.0mm from Bregma - primarily visual cortex (VC) regions). Middle, reference brain outline image with ROIs. Right, the contralateral (CL) traces from the same area (solid green ROI). The somatosensory (SS) area is outlined. (**B**) Left, IL traces for HbT, HbO and HbR from the IL ROI highlighted in dotted green in the middle image (at Bregma - primarily hind paw (HP) somatosensory regions). Middle, reference brain outline image with ROIs. Right, the CL traces from the same area (solid green ROI). HP regions of the SS cortex are highlighted (blue lines) as well as the SS cortex (grey outline). (**C**) Left, traces for HbT, HbO and HbR from the IL ROI highlighted in dotted green in the middle image (+2.0mm from Bregma - primarily somatomotor cortex regions). Middle, reference brain outline with ROIs at this location. Right, CL traces from the same area (solid green ROI). In all cases no activity like that seen during electrical paw stimulation is observed with fluctuations likely resulting from resting state changes. The response from a single cycle from each mouse is plotted as a lightly dotted line in all cases. The bold line represents the averaged response from all mice. Legend - IL: Ipsilateral, CL: Contralateral, HP: Hindpaw region of somatosensory cortex, SS: Somatosensory cortex, Br: Brain Outline. The average response from all trials from each mouse is plotted as a lightly dotted line in all cases. The bold line represents the averaged response from all mice.
